# Hepatoprotective Effect of Silymarin (*Silybum marianum*) on Hepatotoxicity Induced by Acetaminophen in *Spontaneously Hypertensive Rats*

**DOI:** 10.1155/2015/538317

**Published:** 2015-03-02

**Authors:** Abel Felipe Freitag, Gabriel Fernando Esteves Cardia, Bruno Ambrósio da Rocha, Rafael Pazzinatto Aguiar, Francielli Maria de Souza Silva-Comar, Ricardo Alexandre Spironello, Renata Grespan, Silvana Martins Caparroz-Assef, Ciomar Aparecida Bersani-Amado, Roberto Kenji Nakamura Cuman

**Affiliations:** Department of Pharmacology and Therapeutics, State University of Maringá, Avenida Colombo 5790, 870020-900 Maringa, PR, Brazil

## Abstract

This study was aimed to investigate the effect of Silymarin (SLM) on the hypertension state and the liver function changes induced by acetaminophen (APAP) in *spontaneously hypertensive rat* (SHR). Animals normotensive (N) or hypertensive (SHR) were treated or not with APAP (3 g/kg, oral) or previously treated with SLM. Twelve hours after APAP administration, plasmatic levels of liver function markers: alanine aminotransferase (ALT), aspartate aminotransferase (AST), glucose (GLU), gamma glutamyl transferase (*γ*-GT), and alkaline phosphatase (ALP) of all groups, were determined. Liver injury was assessed using histological studies. Samples of their livers were then used to determine the myeloperoxidase (MPO) activity and nitric oxide (NO) production and were also sectioned for histological analysis. No differences were observed for ALT, *γ*-GT, and GLU levels between SHR and normotensive rats groups. However, AST and ALP levels were increased in hypertensive animals. APAP treatment promoted an increase in ALT and AST in both SHR and N. However, only for SHR, *γ*-GT levels were increased. The inflammatory response evaluated by MPO activity and NO production showed that SHR was more susceptible to APAP effect, by increasing leucocyte infiltration. Silymarin treatment (Legalon) restored the hepatocyte functional and histopathological alterations induced by APAP in normotensive and hypertensive animals.

## 1. Introduction

A growing number of patients require several drugs to treat multiple chronic disorders. The prescription of multiple drugs is related to increase risk of adverse drug-related events, among these, hepatic injury. The liver is of vital importance in intermediary metabolism and is continuously exposed to xenobiotics, environmental pollutants, and chemotherapeutic agents, since it is involved in detoxification and elimination of toxic substances [1]. Hepatic damage is associated with altered metabolic functions and it is still a severe health problem, since conventional drugs used in the treatment of liver diseases have serious adverse effects [2].

Cardiovascular diseases such as arterial hypertension (AH) promote functional and structural changes in blood vessels and myocardium, which lead to left ventricular hypertrophy, myocardial infarction, cerebral vascular accident, renal disease, and complications of vital organs [2–4]. Furthermore, AH promotes changes in blood flow and can influence the blood perfusion of different organs, such as kidneys, lungs, and liver. Thus, hypertensive patients could develop severe hepatic damage and several experimental hepatotoxicity models are used to investigate new pharmacologic treatment. However, there are few studies investigating the new strategic therapeutic in hypertensive animals with hepatotoxicity.

APAP is widely used as analgesic-antipyretic drug and is considered remarkably safe drug when used at usual therapeutic doses. APAP toxicity is due to the formation of toxic metabolites when a part of it is metabolized by cytochrome P450 [5]. It is metabolized by sulfation and glucuronidation of the parahydroxyl group. APAP hepatotoxicity is caused by its reactive metabolite* N*-acetyl-p-benzoquinone imine (NAPQI), which causes oxidative stress and glutathione (GSH) depletion, a prerequisite for APAP-induced hepatotoxicity [6, 7]. In overdoses, it is a potent hepatotoxin, producing fulminant hepatic and renal tubular necrosis, which can be lethal in human and animal. Several studies about protection against hepatotoxicity have been investigated to ameliorate the livers disorders treatment. Many formulations containing herbal extracts are used for regeneration of hepatic cells and for protection of the liver against damage [8]. Silymarin (SLM) is a lipophilic extract isolated from the seeds and fruits of* Silybum marianum*, a herbaceous plant belonging to the family Compositae and native to a narrow area of the Mediterranean. SLM is composed of several flavonolignans isomers (65–80%) with small amounts of flavonoids and fatty acids (20–35%) and other polyphenolic compounds [9–11]. The main isolated and structural active component of SLM is silybin, comprising about 33% of total SLM weight, and is clinically used [12, 13] as hepatoprotector to treat liver injuries [14]. Besides, other biological activities for SLM, such as hepatoprotective/hepatic regenerator, immunomodulator, anti-inflammatory, antioxidant, and antifibrotic activities were described [11, 13, 15]. There is evidence that SLM is effective in hepatic disease induced by different drugs [14, 16]. Currently, SLM is used as a reference drug in the screening of new drugs hepatoprotective [11, 17, 18]. This study aimed to investigate the effect of Silymarin on changes in the liver function after APAP administration in SHR, because cardiovascular diseases (including AH) which promote structural and functional changes in blood vessels and myocardium [19] may interfere in the blood perfusion of different organs, such as kidney, lungs, heart, and also liver.

## 2. Materials and Methods

### 2.1. Animals

Normotensive Wistar male rats (N, diastolic = 95 ± 2 mmHg, systolic = 124 ± 5 mmHg) and SHR (*spontaneously hypertensive rat*, diastolic = 191 ± 2 mmHg, systolic = 231 ± 1 mmHg) [20], aged 14–16 weeks, weighing 250–330 g were provided by the Central Animal House of the State University of Maringá. The animals were housed at 23 ± 2°C under a 12/12 h light/dark cycle water and ration (Nuvilab)* ad libitum*. Prior the experiments, the animals were during overnight 12 h before APAP administration, water provided* ad libitum*. The experimental protocols were approved by the Ethical Committee in Animal Experimentation of the State University of Maringá (144/2012 CEAE/UEM).

### 2.2. Hepatotoxicity Induced by APAP and Treatment of Animals with Silymarin (SLM)

The animals were divided into 6 experimental groups of 12 animals each. The N and SHR received orally vehicle (saline containing 2% Tween 80), APAP (3 g/kg) [21] or APAP + SLM (200 mg/kg), before APAP-induced hepatotoxicity as described [22].

### 2.3. Determination of Serum AST, ALT, GLU, *γ*-GT, and ALP Levels

After 12 hours of hepatotoxicity induced by APAP, all rats were anesthetized with halothane 3% and blood was collected from inferior vena cava for determination of plasmatic ALT (alanine aminotransferase), AST (aspartate aminotransferase), GLU (glucose), *γ*-GT (gamma glutamyl transferase), and ALP (alkaline phosphatase) using the Analyze Gold Kits.

### 2.4. Determination of MPO (Myeloperoxidase) Activity

The MPO enzyme activity was measured in the supernatant of homogenate of liver tissue sections. Briefly, the liver sections were put in phosphate buffered saline (PBS) in a Potter homogenizer and the homogenate was stirred in a vortex and centrifuged. Ten microliters of the supernatant was added to each well in triplicate in a 96-well microplate. The PBS solution (200 *μ*L) that contained 4.21 mg o-dianisidine dihydrochloride (Sigma), 22.5 mL double-distilled water, 2.5 mL potassium phosphate buffer (pH = 6), and 10 *μ*L of 1% H_2_O_2_ was added. The enzyme reaction was stopped by 30 *μ*L the addition of sodium acetate (2.23 g in 20 mL of double-distilled water). Enzyme activity was determined by the absorbance measured at 450 nm using a microplate spectrophotometer (Asys Expert Plus).

### 2.5. Determination of NO (Nitric Oxide) Production

The NO production was determined by the Griess method in the supernatant of liver tissue sections, which determines the nitrite production [23]. Two hundred microliters of the supernatant was added to each well in triplicate in a 96-well microplate. Sequentially, solution (50 *μ*L) was added to Griess (1 g sulfanilamide in 2.5 mL phosphoric acid and 0.1 g dihydrochloride of N-(1-naphtyl)ethylenediamine milli-Q water) at room temperature. The reading was taken using an ELISA plate reader at a wavelength of 550 nm. ON production was calculated from a standard curve of sodium nitrite. The results were expressed as *μ*M.

### 2.6. Liver Index and Histopathological Analysis

The livers of rats were collected and inspected macroscopically. The liver index was calculated as liver weight divided by body weight. The largest right lobe of each liver was excised and fixed in a 10% formalin solution for histopathologic analyses of all animals. Subsequently, the livers were dehydrated in increasing concentrations of alcohol (80–100%, v/v) and embedded in paraffin blocks which were sectioned in 6 *μ*m thickness on a Leica Rotary Microtome (Leica Microsystems, Gladesville, NSW, Australia). The organ sections were stained with hematoxylin/eosin (H&E) for evaluation of tissue morphology using light microscopy. The changes in tissue morphology were assessed for nuclear variations, cytoplasmic eosinophilia, swelling, and vacuolation in both periportal and central areas. Percentage of hepatic lesion area was estimated as [inflammatory area/total area] × 100 [24].

### 2.7. Statistical Analysis

Data were expressed as the mean ± SEM for each group. Results were statistically analyzed by using one-way variance analysis (ANOVA) followed by Tukey's test. Differences were considered significant when *P* < 0.05. Statistical analyses were performed using GraphPad Prism (Version 5.0 GraphPad Software, Inc.). Results are expressed and represented in separate experiments.

## 3. Results

The SLM effects on liver weight in rats with APAP-induced hepatotoxicity are presented in Table 1. Significant differences in the liver weight were observed after hepatotoxicity induced by APAP or pretreatment with the SLM, in both groups. The liver index was increased after APAP treatment but restored to normal values after SLM pretreatment (Table 1).

**Figure 1:**
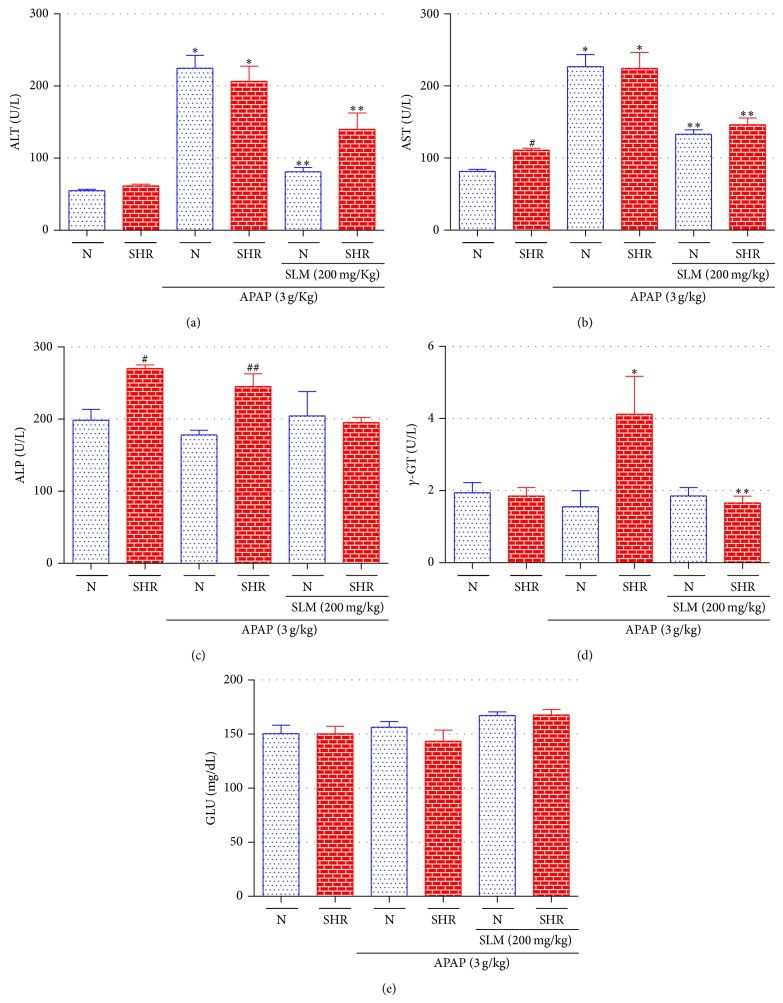
Serum parameters of rats of all groups untreated, treated with APAP (3 g/kg, orally), and pretreated with SLM (200 mg/kg) of ALT (a), AST (b), ALP (c), *γ*-GT (d), and GLU (e) were determined 12 h after APAP intoxication. Results represent mean ± SEM of 12 rats per group. ^*^ *P* < 0.05 N + APAP versus N, ^**^ *P* < 0.01 N + SLM + APAP versus N + APAP, ^*^ *P* < 0.05 SHR + APAP versus SHR, ^**^ *P* < 0.05 SHR + SLM + APAP versus SHR + APAP, ^#^ *P* < 0.05 N versus SHR, and ^##^ *P* < 0.05 N + APAP versus SHR + APAP.

The effects of SLM on plasmatic ALT, AST, ALP, *γ*-GT, and GLU were investigated after APAP treated rats and were represented in Figure 1. Data did not show a significant difference in plasmatic ALT levels between N and SHR animals. However, a significant increase (72% and 71%, resp.) in ALT levels after APAP treatment, in both groups, was verified, whereas SLM treatment was restored (64% and 58%) to normal levels (Figure 1(a)).

Data showed a significant difference in plasmatic AST levels between N and SHR animals. However, a significant increase (61% and 54%, respectively) in AST levels after APAP treatment in both groups, which was restored by SLM (40% and 42%) (Figure 1(b)). In the same manner, significant difference in ALP levels were observed when compared SHR and N groups. However thus altered levels after SLM treatment (Figure 1(c)).

The levels of *γ*-GT (Figure 1(d)) were increased in SHR + APAP treatment (55%) when compared to that of SHR, which was restored by SLM treatment (61%). Therefore, significant differences were not found for APAP treatment and SLM pretreatment in both groups to GLU levels (Figure 1(e)).

**Figure 2:**
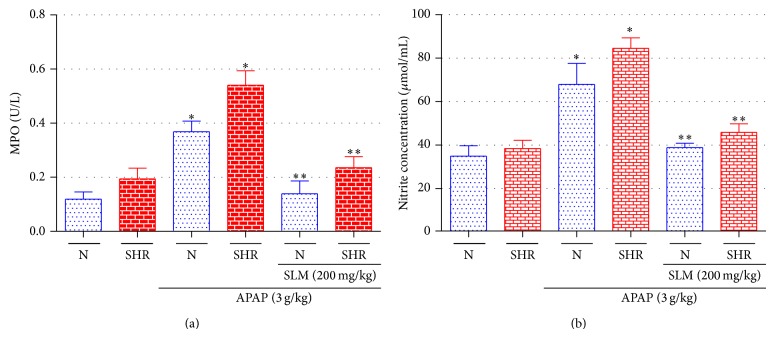
Serum parameters of rats of all groups untreated, treated with APAP (3 g/kg, orally), and pretreated with SLM (200 mg/kg) of MPO activity (a) and NO production (b) were determined 12 h after APAP intoxication. Results represent mean ± SEM of 12 rats per group. ^*^ *P* < 0.05 N + APAP versus N, ^**^ *P* < 0.05 N + SLM + APAP versus N + APAP, ^*^ *P* < 0.05 SHR + APAP versus SHR, and ^**^ *P* < 0.05 SHR + SLM + APAP versus SHR + APAP.

The effects of SLM on MPO activity and NO production in liver tissues were investigated after APAP treated rats and were represented in Figure 2. The MPO activity in SLM pretreated rats was significantly decreased in N (64%) and SHR (57%), when compared with that of APAP group (increased of 69% in N and 65% in SHR) (Figure 2(a)).

In Figure 2(b) the N and SHR animals group treated with an overdose of APAP developed significant hepatic damage, which was observed by a substantial increase in the NO production. Administration of SLM after APAP treatment resulted in a significant reduction (43% in N and 46% in SHR) in NO production in APAP groups (49% and 54%, resp.) and appears to be protective in reducing the injurious effect of APAP.

**Figure 3:**
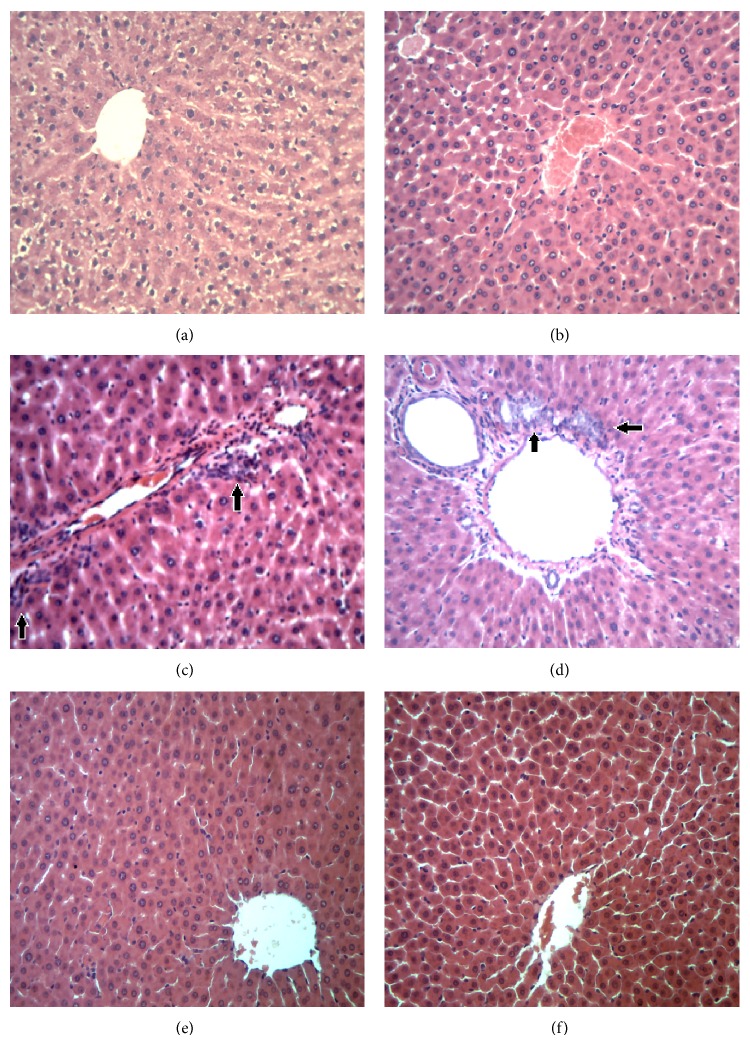
Histopathology of livers 12 hours after APAP injection. The livers were collected 12 hours from all groups after APAP administration (3 g/kg). Panels (a) and (b): N and SHR group that received only vehicle, respectively, (c) and (d): N and SHR group that received APAP, respectively. (e) and (f): N and SHR group pretreated with standard drug (SLM, 200 mg/kg per 7 days). Arrows indicate leukocyte infiltration. Sections were stained with H&E (magnification, ×40).

The livers of untreated SHR and N groups did not show any histopathological alteration (Figures 3(a) and 3(b)), whereas the treatment with APAP showed severe injury characterized by features typical of inflammatory hepatic tissues, including the presence of moderate infiltration of neutrophils and inflammation, characteristics of hepatic damage as indicated by biochemical and enzymatic assays (Figures 3(c) and 3(d)). After SLM treatment, by hystopatological analysis it was demonstrated that no pathological hallmark were observed after induced by APAP treatment (Figures 3(e) and 3(f)).

Liver histopathology also revealed an increased inflammatory area in APAP normotensive and hypertensive rats (Figures 3(c) and 3(d): 37.7% and 48.9%, resp.) at 12 h compared with rats that received orally APAP vehicle (Figures 3(a) and 3(b)). Morphometric analysis of hepatic lesions at 12 h indicated a reduction in the inflammatory area percentage in N and SHR rats pretreated with SLM (Figures 3(e) and 3(f): 31.7% and 36.7%, resp.) when compared with APAP treatment.

## 4. Discussion and Conclusion

Chronic patients often are treated with multiple drugs. During hypertensive state many structural and functional alterations occur, such as: increase in the blood flux, organs perfusion and ROS release related to oxidative stress burst. Thus, it is plausible to assume that hypertensive state could enhance the hepatic damage and other injuries in various organs.

The liver is involved in detoxification and elimination of drugs and a liver injury could affect pharmacokinetics parameters of drugs and increase the risk of adverse drug effects.

Liver injuries induced by APAP is commonly used as a model for the screening of hepatoprotective activities of drugs, where free radicals and oxidative processes play an important role in hepatotoxicity [8].

A decrease in total serum protein after APAP treatment could be associated with the decrease in the number of hepatocytes, which in turn may result in the decreased hepatic capacity to synthesize protein and, consequently, decrease liver weight [25, 26]. Our results showed that livers weight of hypertensive and normotensive rats did not differ. However, changes are observed in the liver index that was increased after APAP treatment but restored to normal values after SLM pretreatment.

Hepatotoxic drugs, such as APAP, are known to cause marked elevation in serum level of enzymes, such as ALT, AST, ALP, and bilirubin, indicating significant hepatocellular injury [27]. When there is hepatopathy, these enzymes leak into the blood stream in conformity with the extent of liver injury [28, 29]. Our data showed that the liver activity functional is altered, since ALT, AST, *γ*-GT, and ALP levels are increased in SHR, but not for glucose levels [30]. Generally, there is a raised activity of serum transaminases in intoxicated rats [31], as observed in the present study. However, ALT and AST levels were increased but this is enough to be attributed to the damaged structural integrity of the liver because transaminases are cytoplasmic enzymes in nature and are released into the circulation after cellular damage [32]. Interestingly, in our work, *γ*-GT levels were increased after APAP treatment, but not in normotensive animals. *γ*-GT is a common biomarker of liver injury and alcohol consumption [33]. However, recent epidemiologic and clinical studies have also found a close association between *γ*-GT level and the risk of cardiovascular disease, diabetes, and metabolic syndrome, demonstrating an association between hypertension and hepatic injury [19, 34, 35], as demonstrated by our results.

Silymarin has hepatoprotective properties and is used in treatment of various liver diseases [11]. Various studies indicate that Silymarin exhibits strong antioxidant activity [36] and shows protective effects against hepatic toxicity induced by a wide variety of agents by inhibiting lipid peroxidation [30, 37]. Higher total phenolic content has been known to contribute to the antioxidant activity of extracts [38], while antioxidant activity has also been linked to the hepatoprotective effect of some extracts [38, 39]. These findings corroborate with our results on the ability of SLM to exert a hepatoprotective activity.

In this study, SLM treatment restore to normal values the levels of ALT, AST in all animals treated with APAP, and also in *γ*-GT levels in SHR, which were altered only in this animal group. The reduced concentrations of these enzymes as a result of SLM administration might probably be, in part, due to the presence of chemical constituents in the extract [17]. The plasmatic reduction of these marker enzymes, to return to near normal values, would be owing to the antihepatotoxic effect of SLM.

Histopathologic studies supported the evidence of biochemical parameters analysed in this study. Histological analyses of rat liver treated with APAP showed significant hepatotoxicity, characterized by inflammatory hepatic tissues, including the presence of moderate infiltration of neutrophils. There was extensive infiltration of inflammatory cells around the central vein and loss of cellular boundaries in all groups, after hepatotoxicity induced by APAP [24]. High activity of MPO and NO production was used to demonstrate an acute inflammatory process as observed after the liver injury induced by APAP. The inflammatory response evaluated by MPO activity and NO production showed that SHR is more susceptible to APAP effect, by increasing the leucocyte infiltration. The NO production, a free radical present in the inflammatory process, did not differ in SHR and N groups. After treatment with APAP there was a significant increase in NO production in both groups of animals. Pretreatment with SLM decreased NO production, suggesting activities anti-inflammatory and anti-free radicals properties of this drug. SLM treatment reduced the severity of hepatic damage, when compared to that observed after APAP treatment, decreasing neutrophil infiltration in the hepatic tissue which further indicated its significant hepatoprotective effect.

In light of our study, here we demonstrated an important association between hypertension and hepatic damage. SLM was effective to protect the liver of damage induced by APAP. The use of natural products as SLM to treated hepatotoxicity could be important to patients receiving several medicines, due to chronic diseases.

## Figures and Tables

**Table 1:** 

Treatment	Body weight (g)	Liver weight (g)	Liver index (%)
N	309 ± 11	11.63 ± 0.42	3.4 ± 0.04
SHR	267 ± 9^#^	11.10 ± 0.15	3.8 ± 0.04
N + APAP	321 ± 6	11.39 ± 0.33	3.7 ± 0.1^*^
SHR + APAP	248 ± 10^##^	10.12 ± 0.36	4.4 ± 0.1^**^
N + SLM + APAP	317 ± 8	11.02 ± 0.37	3.5 ± 0.07^*^
SHR + SLM + APAP	233 ± 4^###^	8.89 ± 0.34	3.4 ± 0.1^**^

Body weight (g), liver weight (g), and liver index (%) in all groups of rats 12 hours after APAP administration (3 g/kg) and pretreated with SLM (200 mg/kg). Media ± SEM, *n* = 12 por grupo. ^*^ *P* < 0.05 N + APAP versus N, ^*^ *P* < 0.01 N + SLM + APAP versus N + APAP, ^**^ *P* < 0.05 SHR + APAP versus SHR, ^**^ *P* < 0.05 SHR + SLM + APAP versus SHR + APAP,  ^#^ *P* < 0.05 N versus SHR, ^##^ *P* < 0.05 N + APAP versus SHR + APAP, and ^###^ *P* < 0.05 N + SLM + APAP versus SHR + SLM + APAP.

## References

[1] Gu X., Manautou J. E. (2012). Molecular mechanisms underlying chemical liver injury. *Expert Reviews in Molecular Medicine*.

[2] Hayden M. R., Sowers J. R. (2008). Treating hypertension while protecting the vulnerable islet in the cardiometabolic syndrome. *Journal of the American Society of Hypertension*.

[3] Plante G. E. (2002). Vascular response to stress in health and disease. *Metabolism: Clinical and Experimental*.

[4] Mambelli E. (2011). Arterial hypertension in dialysis: up to what point should it be corrected? It depends. *GiornaleItaliano di Nefrologia*.

[5] Das J., Ghosh J., Manna P., Sil P. C. (2010). Acetaminophen induced acute liver failure via oxidative stress and JNK activation: protective role of taurine by the suppression of cytochrome P450 2E1. *Free Radical Research*.

[6] Aubert J., Begriche K., Delannoy M. (2012). Differences in early acetaminophen hepatotoxicity between obese ob/ob and db/db mice. *Journal of Pharmacology and Experimental Therapeutics*.

[7] Zhang L., Gavin T., Geohagen B. C., Liu Q., Downey K. J., LoPachin R. M. (2013). Protective properties of 2-acetylcyclopentanone in a mouse model of acetaminophen hepatotoxicity. *Journal of Pharmacology and Experimental Therapeutics*.

[8] Jaeschke H., Williams C. D., McGill M. R., Xie Y., Ramachandran A. (2013). Models of drug-induced liver injury for evaluation of phytotherapeutics and other natural products. *Food and Chemical Toxicology*.

[9] Kim N.-C., Graf T. N., Sparacino C. M., Wani M. C., Wall M. E. (2003). Complete isolation and characterization of silybins and isosilybins from milk thistle (*Silybum marianum*). *Organic & Biomolecular Chemistry*.

[10] Lee J. I., Hsu B. H., Wu D., Barrett J. S. (2006). Separation and characterization of silybin, isosilybin, silydianin and silychristin in milk thistle extract by liquid chromatography-electrospray tandem mass spectrometry. *Journal of Chromatography A*.

[11] Elmowafy M., Viitala T., Ibrahim H. M. (2013). Silymarin loaded liposomes for hepatic targeting: in vitro evaluation and HepG2 drug uptake. *European Journal of Pharmaceutical Sciences*.

[12] Pradhan S. C., Girish C. (2006). Hepatoprotective herbal drug, silymarin from experimental pharmacology to clinical medicine. *Indian Journal of Medical Research*.

[13] Fehér J., Lengyel G. (2012). Silymarin in the prevention and treatment of liver diseases and primary liver cancer. *Current Pharmaceutical Biotechnology*.

[14] Hau D. K.-P., Wong R. S.-M., Cheng G. Y.-M. (2010). Novel use of silymarin as delayed therapy for acetaminophen-induced acute hepatic injury. *Forschende Komplementarmedizin*.

[15] Das S., Roy P., Auddy R. G., Mukherjee A. (2011). Silymarin nanoparticle prevents paracetamol-induced hepatotoxicity. *International Journal of Nanomedicine*.

[16] Sherif I. O., Al-Gayyar M. M. H. (2013). Antioxidant, anti-inflammatory and hepatoprotective effects of silymarin on hepatic dysfunction induced by sodium nitrite. *European Cytokine Network*.

[17] Cordero-Pérez P., Torres-González L., Aguirre-Garza M. (2013). Hepatoprotective effect of commercial herbal extracts on carbon tetrachloride-induced liver damage in Wistar rats. *Pharmacognosy Research*.

[18] Raj S., Gothandam K. M. (2014). Hepatoprotective effect of polyphenols rich methanolic extract of *Amorphophallus commutatus* var. *wayanadensis* against CCl__4__ induced hepatic injury in swiss albino mice. *Food and Chemical Toxicology*.

[19] Houschyar K. S., Lüdtke R., Dobos G. J. (2012). Effects of phlebotomy-induced reduction of body iron stores on metabolic syndrome: results from a randomized clinical trial. *BMC Medicine*.

[20] Salsoso R., Guzmán-Gutiérrez E., Arroyo P. (2014). Reduced L-carnitine transport in aortic endothelial cells from spontaneously hypertensive rats. *PLoS ONE*.

[21] Fakurazi S., Hairuszah I., Nanthini U. (2008). *Moringa oleifera* Lam prevents acetaminophen induced liver injury through restoration of glutathione level. *Food and Chemical Toxicology*.

[22] Quan J., Yin X., Xu H. (2011). Boschniakia rossica prevents the carbon tetrachloride-induced hepatotoxicity in rat. *Experimental and Toxicologic Pathology*.

[23] Silvia Fröde Saleh T., Batista Calixto J., Santos Medeiros Y. (1999). Effects of anti-inflammatory drugs upon nitrate and myeloperoxidase levels in the mouse pleurisy induced by carrageenan. *Peptides*.

[24] Singhal R., Ganey P. E., Roth R. A. (2012). Complement activation in acetaminophen-induced liver injury in mice. *Journal of Pharmacology and Experimental Therapeutics*.

[25] Bhadauria M. (2010). Dose-dependent hepatoprotective effect of emodin against acetaminophen-induced acute damage in rats. *Experimental and Toxicologic Pathology*.

[26] Oyagbemi A. A., Odetola A. A. (2010). Hepatoprotective effects of ethanolic extract of Cnidoscolus aconitifolius on paracetamol-induced hepatic damage in rats. *Pakistan Journal of Biological Sciences*.

[27] Green T. J., Sivilotti M. L. A., Langmann C. (2010). When do the aminotransferases rise after acute acetaminophen overdose. *Clinical Toxicology*.

[28] Alkiyumi S. S., Abdullah M. A., Alrashdi A. S., Salama S. M., Abdelwahab S. I., Hadi A. H. A. (2012). *Ipomoea aquatica* extract shows protective action against thioacetamide-induced hepatotoxicity. *Molecules*.

[29] Bell L. N., Vuppalanchi R., Watkins P. B. (2012). Serum proteomic profiling in patients with drug-induced liver injury. *Alimentary Pharmacology and Therapeutics*.

[30] Binda D., Nicod L., Viollon-Abadie C. (2001). Strain difference (WKY, SPRD) in the hepatic antioxidant status in rat and effect of hypertension (SHR, DOCA). Ex vivo and in vitro data. *Molecular and Cellular Biochemistry*.

[31] Dremza I. K., Cheshchevik V. T., Zabrodskaya S. V. (2010). Hepatotoxic efects of acetaminophen. Protective properties of tryptophan-derivatives. *Biomeditsinskaya Khimiya*.

[32] Kelava T., Ćavar I., Vukojević K., Saraga-Babić M., Čulo F. (2013). The effect of glucagon and cyclic adenosine monophosphate on acute liver damage induced by acetaminophen. *Histology and Histopathology*.

[33] Nakanishi N., Suzuki K., Tatara K. (2004). Serum *γ*-glutamyltransferase and risk of metabolic syndrome and type 2 diabetes in middle-aged Japanese men. *Diabetes Care*.

[34] Ryu S., Chang Y., Woo H. Y. (2010). Longitudinal increase in (gamma)-glutamyltransferase within the reference interval predicts metabolic syndrome in middle-aged Korean men. *Metabolism: Clinical and Experimental*.

[35] Lee D. S., Evans J. C., Robins S. J. (2007). Gamma glutamyl transferase and metabolic syndrome, cardiovascular disease, and mortality risk: the Framingham Heart Study. *Arteriosclerosis, Thrombosis, and Vascular Biology*.

[36] Simeonova R., Vitcheva V., Kondeva-Burdina M., Krasteva I., Manov V., Mitcheva M. (2013). Hepatoprotective and antioxidant effects of saponarin, isolated from *Gypsophila trichotoma* wend. on paracetamol-induced liver damage in rats. *BioMed Research International*.

[37] Bosisio E., Benelli C., Pirola O. (1992). Effect of the flavanolignans of *Silybum marianum* L. on lipid peroxidation in rat liver microsomes and freshly isolated hepatocytes. *Pharmacological Research*.

[38] Yuan L., Gu X., Yin Z., Kang W. (2014). Antioxidant activities in vitro and hepatoprotective effects of *Nelumbo nucifera* leaves in vivo. *African Journal of Traditional, Complementary and Alternative Medicines*.

[39] Gu F., Gu X., Xu Q., Kang W. (2014). Antioxidant activity in vitro and hepatoprotective effect of *Phlomis maximowiczii* in vivo. *African Journal of Traditional, Complementary, and Alternative Medicines*.

